# Improved patient-reported outcomes after interprofessional training in mental health: a nonrandomized intervention study

**DOI:** 10.1186/s12888-020-02616-x

**Published:** 2020-05-14

**Authors:** Michael Marcussen, Birgitte Nørgaard, Karen Borgnakke, Sidse Arnfred

**Affiliations:** 1 Department of Clinical Medicine, University of Copenhagen and Psychiatry Slagelse, Region Zealand Mental Health Service, Fælledvej 6, 4200 Slagelse, Denmark; 2 Department of Public Health, University of Southern Denmark, Odense, Denmark; 3 Department of Media, Cognition and Communication, University of Copenhagen, Copenhagen, Denmark

**Keywords:** *Patient-reported outcomes, *PRO, *Interprofessional training, *IPE, *Team-based care, *Mental health services, *Inpatients

## Abstract

**Background:**

Collaborative interprofessional practices are essential in caring for people with complex mental health problems. Despite the difficulties of demonstrating positive impacts of interprofessional education (IPE), it is believed to enhance interprofessional practices. We aimed to assess impacts on patient satisfaction, self-reported psychological distress and mental health status in a psychiatric ward.

**Methods:**

We conducted a nonrandomized intervention study with patient satisfaction, psychological distress, and health status as outcomes. Mental health inpatients were referred to either an interprofessional training unit (intervention group) or to a conventionally organized ward (comparison group). Outcomes were assessed using the Short Form Health Survey (SF-36), the Kessler Psychological Distress Scale (K10), and the Client Satisfaction Questionnaire (CSQ-8).

**Results:**

The intervention group included 129 patients, the comparison group 123. The former group reported better mental health status than the latter; the postintervention mean difference between them being 5.30 (95% CI 2.71–7.89; *p* = 0.001; SF-36), with an effect size of 0.24. The intervention group patients also scored higher on satisfaction (mean difference 1.01; 95% CI 0.06–1.96; *p* = 0.04), with an effect size of 0.31. The groups’ mean scores of psychological distress were identical.

**Conclusion:**

Our results support the hypothesized value of interprofessional training: intervention group patients reported higher scores regarding mental health status and satisfaction than did comparison group patients. As IPE interventions have rarely involved patients and fewer have taken place in practice settings, further research into both the processes and the long-term effects of IPE in mental healthcare is needed.

**Trial registration:**

The study was registered in ClinicalTrials.gov: NCT03070977 on March 6, 2017.

## Background

The importance of professional teamwork to prevent relapse and manage chronic conditions [1, 2] in patients with severe mental health disorders is generally acknowledged. As all aspects of the patient’s life are affected [1–3], the treatment requires participation from a diversity of healthcare professionals working together in specialized teams [2, 4, 5]. However, collaboration among team members is often fraught with problems, affecting the quality of care in terms of poor service delivery, low patient satisfaction, and errors [1, 2, 5, 6]. Since the 1990s interprofessional collaboration has been promoted and endorsed internationally as a means to improve mental healthcare [1]. Interprofessional education (IPE) is assumed to enhance such collaborative practices, although the field has met challenges in demonstrating effects on mental health outcomes [2, 7]. Policymakers nevertheless continue to invoke IPE as a way to improve collaboration, and to call for its wider implementation across educational and clinical settings [1, 6]. The World Health Organization (WHO) defines IPE as settings in which “(…) students from two or more professions learn about, from and with each other to enable effective collaboration and improve health outcomes” [6]. According to the literature, teams generate better patient-outcomes [2, 8], stimulate communication and partnership among professionals and patients [1, 9]. Improved patient satisfaction, an increasingly important and commonly used indicator for measuring the quality of care [10, 11], is also reported [8, 12].

Despite the patients’ obvious stake in healthcare outcomes and interest in participation in research aimed at improving practice [13], their perspectives have been sought by only a few IPE initiatives [14, 15]. The outcomes of mental health research have traditionally been assessed through objectively measured clinical information, such as relapse rates, hospitalization, and the degree of symptom reduction [16]. However, an increasing number of intervention studies include self-reported measures of well-being, focusing on the patients’ perceptions of their health-related quality of life or health status [17, 18]. Despite the proliferation of teamwork in mental health, its association with improved patient-reported outcomes, such as patient satisfaction, is poorly documented [19]. Although this field is relatively sparsely researched in comparison to that of physical health, there is convincing evidence in favour of shared decision-making to underpin mental health treatment [20, 21]. Involving the patients in the treatment is vital, as they are in the best position to value its effectiveness [11]. Moreover, interventions based on interprofessional care and a patient-centred approach have been shown to improve patient-reported outcomes and satisfaction in mental healthcare [13, 22]. Hence, we aimed to investigate the impact of students’ interprofessional training on patient satisfaction, self-reported psychological distress and mental health status.

An interprofessional clinical training unit was established in a psychiatric ward for students from the following professions: medicine, nursing, pedagogy, physiotherapy, and social work. Inspired by the work of Nørgaard et al., the organization of the ward allowed the students to learn from each other while developing competences in interprofessional collaboration [23]. Work in the unit followed IPE precepts, with students participating in team-based care [24, 25]. The team organization aimed to ensure coordinated treatment and care, and to accomplish shared treatment goals. In the 9 months leading up to the intervention, we conducted a pilot project to test the intervention and its measurements. The pilot aimed at strengthening and assessing the training unit concept. Furthermore, the pilot period showed positive preliminary results after conceptualization of the intervention involving 44 patients (July–September 2016). In comparison with the patients in the standard ward, the training unit patients scored higher on mental health status (28.2 and 31.1, respectively; adjusted mean difference 2.9). Therefore, we expected improved patient-reported outcomes for the intervention group patients. It was hypothesized that the IPE intervention would be associated with a bigger improvement of patient-reported outcomes and satisfaction at hospital discharge than conventional clinical practice.

## Methods

### Design

We designed a nonrandomized intervention study with an intervention group (in the interprofessional training unit) and a comparison group (in the conventional inpatient ward). Patients were recruited between October 2016 and March 2018. After initial admission to the emergency ward, the patients were referred to inpatient wards based on their home address. The two wards were comparable in terms of patients’ diagnoses, staffing, and physical layout (17 single-bed rooms). Questionnaires were administered to both groups at admission (T1) and at discharge (T2). The design enabled the comparison of change over time. The study was retrospectively registered in ClinicalTrials.gov: NCT03070977 on March 6, 2017 and adhered to the CONSORT guidelines [26].

### Setting

The study took place at the Psychiatric Hospital in Slagelse, Denmark, which consists of four inpatient wards, an outpatient clinic, and an emergency ward serving a mixed urban and rural district. The department of psychiatry has 80 beds, 1995 discharges, and 39,391 visits to its outpatient clinics per year (2017 figures, obtained from HR department, Mental Health Services, Slagelse). As part of the publicly funded hospital services, the mental health services are administered by Region Zealand, one of Denmark’s five regional health authorities, serving a population of 821,000.

### Intervention

#### Interprofessional training unit

Established in 2015, the psychiatric ward was organized into three care teams, each with professionals and students from medicine, nursing, nursing assistants, pedagogy, physiotherapy and social work, supplemented by patients. The training aimed at strengthening the students’ uniprofessional roles, their knowledge of other professionals’ roles, and supporting interprofessional collaboration. Each team was charged with five or six patients. To offer adequate treatment of the inpatients’ complex pathology a complete team was typically required and also for students’ instruction. Furthermore, both students and permanent staff took part in daily work in the ward, such as training, psycho-educational interventions, medication, and other occupation-specific tasks. Three to 10 students were assigned to each team. Representing as many professions as possible, a range of professionals were assigned to the teams. Each team was charged with five or six patients. The inpatients’ complex pathology typically would require a complete care team to offer adequate treatment. An interprofessional collaboration and training course was organized in mid-2016 by the facilitation team responsible for the interprofessional training of students. The intervention involved the entire staff in an initial one-day interactive workshop to facilitate reflection and small-group work focusing on team-based and patient-centred care. Two types of activities were involved in students’ clerkship course: clinical care teamwork (supervised by instructors from the participating professions) and interprofessional group tutorials planned by the facilitation team and led by instructors with extensive experience in delivering IPE.

### Interprofessional group tutorials

To stimulate reflection on clinical practice [5] and to strengthen their knowledge of the patients’ treatment and care, all students met once a week for the interprofessional group tutorials. The students additionally participated in morning and evening shifts attended by supervisors from each of the participating professions to ensure patient safety and an optimal learning environment.

During the day shifts, the students’ clinical training was organized in clinical care teams of variable sizes (3 to 10 students), while in the evening, three or four students were assigned to each team.

### Clinical care team

The patients’ active participation in the teamwork was a key feature of the intervention, which emphasized collaboration between patients and professionals/students in the development of patient-centred team care. At the start of the hospitalization, the goals of the treatment were thus agreed on by the patient and the team. Weekly team conferences were held to ensure the patients’ progress and, if required, to adjust their treatment plans [25]. Patients who declined to take an active role in the treatment continued to be allocated to the intervention group.

### Comparison

The comparison group patients were admitted to a standard psychiatric ward that offered uniprofessional care, in which traditional rounds led by a psychiatrist and supported by registered nurses and nursing assistants were held. Likewise, the students in the comparison group received traditional uniprofessional training during their clinical placement, with no structured interprofessional training. Although students of healthcare all receive their clinical education in the same wards, and are involved with the same patients, their programs are rarely coordinated. With each profession being responsible for the supervision and instruction of students for the development of profession-specific skills, training in the clinic is uniprofessionally organized, as opposed to the team organization in the intervention group.

### Participants and procedure

The study included inpatients admitted from October 2016 to March 2018. Aged 18—65, the patients suffered from psychiatric disorders such as schizophrenia, psychosis, major depression, bipolar disorder, and severe personality disorder. Self-report questionnaires were administered to both patient groups within the first 48 h of their stay in the stationary wards (T1) and on the day of discharge (T2). In addition to those who did not consent to participation in the study, patients were excluded if they failed to complete the questionnaire, had been hospitalized for less than a week, or if clinical staff considered them too ill at admission to complete the survey.

### Outcome measures

#### Health status and psychological distress

The participants’ health status was assessed using the standardized Short Form Health Survey (SF-36), which is widely used to assess physical and mental health. We applied the acute version, with one-week recall [18]. On the basis of the questionnaire’s 36 items, we calculated two summary scores; the physical component score and the mental component score (PCS and MCS, respectively) [27]. All of the eight SF-36 scales contribute in different proportion to the scoring of both PCS and MCS measures. The physical component score was calculated, as we expected no difference in the PCS between the two groups before and after the intervention.

Scores range from 0 (zero) to 100, with higher scores indicating better health. We also assessed nonspecific psychological distress using the Danish version of the Kessler Psychological Distress Scale (K10) [28] with a 4 week recall-period (no acute version is currently available). Validated and culturally adapted by Thelin et al. [29], its 10 items measure the experienced level of anxiety and depressive symptoms over the preceding 4 weeks, with a score range from 10 to 50, higher scores indicating more anxiety and stronger depressive symptoms.

The different recall-periods for SF-36 (1 week) and K10 (4 weeks) were handled by informing the patients to answer the questionnaire with an overall recall-period of 1 week.

#### Patient satisfaction

The patients’ satisfaction was assessed using the 8-item version of the Client Satisfaction Questionnaire (CSQ-8). The questionnaire has been validated in a Danish population [30], and is widely used to measure satisfaction related to care [10]. Items are scored on a Likert scale from 1 to 4, with descriptors for each response point. Total scores range from 8 to 32, with higher scores indicating greater satisfaction. The CSQ-8 has been found to have high internal consistency and concurrent validity in mental health settings [31].

### Data analysis

The trial was powered at 80% (α = 0.05) to detect an effect size of 0.4 in SF-36 score, which we regard as adequate to determine clinically meaningful differences between interventions, and furthermore we expected a relatively high score because of preliminary results from the pilot study. Based on the sample size calculation, 120 participants were needed per group [27]. The participants were described in terms of sex, age, and baseline scores (SF-36 and K10). All scales were tested for internal reliability, and Cronbach’s alpha was estimated at 0.88 (CSQ-8), 0.90 (K10), and 0.73 (SF-36) in overall reliability, which is generally considered acceptable [32]. We applied unpaired t-tests to assess mean score differences at baseline, and chi-square tests for sex distribution. Differences over time were explored using paired sample t-tests. Applying Cohen’s d, effect sizes were derived from calculating mean difference and standard deviations [33]. In order to assess differences in outcomes between groups, we employed linear mixed regression. All statistical analyses were performed using SPSS (IBM Corp. Released 2018. IBM SPSS Statistics for Windows, Version 25.0. Armonk, NY: IBM Corp).

### Ethical considerations

Before participation the patients were informed of the project and its purpose, both verbally and in writing. Response to the questionnaires constituted voluntary consent to participation; this applied for both baseline and follow-up. All patients were invited to participate, including forensic patients and patients admitted under a restraining order, in accordance with the Danish Mental Health Act. If the patients declined to participate in the survey, they were still assigned to the inpatient ward they were referred to by the emergency ward. Data were entered into the EasyTrial© Online Clinical Trial Management system. All personal identifiers were removed or disguised during analysis to preclude personal identification. The authors assert that all procedures contributing to this work comply with the ethical standards of the relevant national and institutional committees and the Helsinki Declaration of 1975, as revised in 2008. The study was approved by the Danish Data Protection Agency (2008-58-0020), and required no further ethical approval according to Danish legislation (16–000014).

## Results

**Fig. 1:**
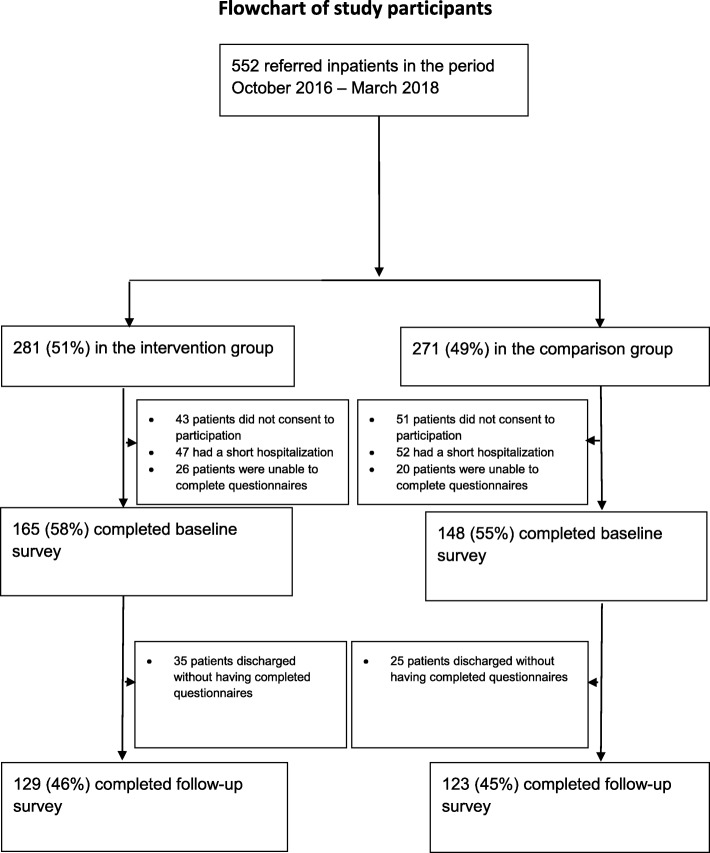
Flow chart of study participants

During the study period, 281 patients were referred to the intervention group, while 271 patients were referred to the comparison group. In Fig. 1, we present the flow of participants through the study: In the intervention group the baseline survey was completed by 164 patients; 129 completed the follow-up; in the comparison group, the corresponding numbers were 148 and 123. The intervention group’s baseline response rate was thus 58%, while 46% completed both baseline and follow-up. In the comparison group the completion rate was 55% for the baseline survey, while 45% completed both baseline and follow-up surveys.

The referrals to the training unit and the standard ward (based on patients’ home address) resulted in two groups of equivalent size. The baseline characteristics of the intervention and the comparison group participants were comparable in terms of gender, age and health status (Table 1).

**Table 1:** Demographic characteristics,health status, and psychological distress at baseline

	T1		
	Intervention (*n* = 165) n (%) + mean + SD	Comparison (*n* = 148) n (%) + mean + SD	P^a^
*Gender*			
Male	87 (52.7)	95 (64.2)	0.05^a^
Female	78 (47.2)	53 (35.8)	
*Age*	39.6; 14.4	40.2; 14.2	0.7^b^
*Baseline scores*			
*K10*	31.7; 8.6	31.6; 8.9	0.9^b^
*SF-36 (PCS)*	47.6; 10.3	48.2; 10.8	0.6^b^
*SF-36 (MCS)*	27.6; 11.5	29.7; 15.1	0.2^b^

*T1* Time 1 measurement (baseline), *T2* Time 2 measurement (at discharge), *SF-36* Short Form Health Survey, *PCS* physical component summary scores, *MCS* mental component summary scores, *K10* Kessler Psychological Distress Scale

^a^ Pearson’s chi-square

^b^ Independent samples t-test

### Health status and psychological distress

We investigated the unadjusted change over time of MCS and K10 scores. The training unit patients scored higher on the MCS, when compared with patients in the standard ward. As seen in Table 2, the unadjusted pre- and post-MCS scores were 27.6--34.2 in the intervention group (*p* = 0.001) and 29.7--30.9 in the comparison group (*p* = 0.1), with an effect size of 0.24. Although baseline adjustment narrowed the range of MCS scores for the intervention group, the range continued to be greater than that of the comparison group (details are given in Table 2). Table 3 shows the adjusted mean difference in MCS scores between the two groups to be 5.30 (95% CI 2.71 to 7.89; *p* = 0.001). The unadjusted physical component summary (PCS) scores ranged between 47.6 and 49.5 for the intervention group (p = 0.1); between 48.2 and 50.5 for the comparison group (*p* = 0.06) (Table 2), with an effect size of 0.10. This difference was nonsignificant, and adjustments did not modify the results (Table 3).

**Table 2:** Outcome measures: Mental Health Status; Psychological distress and patient satisfaction scores at admission and discharge

	Participants							
	T1^a^			T2^b^				
	Intervention (*n* = 165) (mean; SD)	Comparison (*n* = 148) (mean; SD)	P	Intervention (*n* = 120) (mean; SD)	Comparison (*n* = 101) (mean; SD)	Intervention P	Comparison P	Effect size (Cohen’s d)
*SF-36*								
*PCS score*	47.6; 10.3	48.2; 10.8	0.6	49.5; 8.6	50.5; 10.5	0.1	0.06	0.10
*MCS score*	27.6; 11.5	29.7; 15.1	0.2	34.2; 13.0	30.9; 14.3	0.001	0.1	0.24
	(*n* = 163)	(*n* = 146)	P	(*n* = 129)	(*n* = 123)	P	P	
*K10 score*	31.7; 8.6	31.6; 8.9	0.9	29.4; 8.3	29.8; 9.0	0.001	0.001	0.05
				(*n* = 111)	(*n* = 104)	P^a^		
*CSQ-8 score*				25.2; 3.3	24.1; 3.7	0.02		0.31

*SD* Standard deviation, *CSQ-8* Client Satisfaction Questionnaire, *SF-36* Short Form Health Survey, *PCS* physical component summary scores, *MCS* mental component summary scores, *K10* Kessler Psychological Distress Scale

^a^ Independent samples t-test

^b^ Paired t-test

**Table 3:** Estimates of SF-36 and K10 over time and between groups; CSQ-8 between groups

	Intervention (*n* = 120) (mean; SE)	Comparison (*n* = 101) (mean; SE)	Between groups (mean diff.)	CI (Difference-CI)	P
*SF-36*					
*PCS score*	50.1; 0.6	49.7; 0.6	0.40	−2.03–1.24	0.6^a^
*MCS score*	35.1; 0.9	28.8; 1.0	5.30	2.71–7.89	0.001
	(*n* = 129)	(*n* = 123)	Between group (mean diff.)	CI (Difference-CI)	P
K10 score	29.40; 0.5	29.73; 0.6	−0.33	−1.9–1.2	0.7^a^
	(*n* = 111)	(*n* = 104)	Between group (mean diff.)	CI (Difference-CI)	P
Total CSQ-8	25.2; 0.3	24.2; 0.3	1.01	0.06–1.96	0.04^b^

*SE* Standard error, *CSQ-8* Client Satisfaction Questionnaire, *SF-36* Short Form Health Survey, *PCS* physical component summary scores, *MCS* mental component summary scores, *K10* Kessler Psychological Distress Scale

^a^ Linear mixed regression. Mean scores in comparison and intervention groups adjusted for baseline MCS scores, age, and sex

^b^ Linear mixed regression. Mean scores in comparison and intervention groups adjusted for age and sex

The mean K10 scores decreased for both groups, as shown in Table 2. For the intervention group the mean pre- and post-K10 scores were 31.7--29.4 (p = 0.001), while they were 31.6–29.8 (p = 0.001) for the comparison group. After adjustment for gender, age and baseline scores, K10 scores remained similar across groups. Details are displayed in Table 3.

### Patient satisfaction

As seen in Table 2, patients in the training unit reported higher satisfaction with their treatment (CSQ-8; 25.2) than did patients in the standard ward (CSQ-8; 24.1) (*p* = 0.02), with an effect size of 0.31. (The data in Table 2 are unadjusted). After adjustment (Table 3), the difference in mean scores between the two groups was 1.01 (95% CI 0.06 to 1.96; *p* = 0.04).

#### Dropout analysis

The population of eligible patients counted 552, with 47% females, with a mean age of 40.8 years. The responding patients’ mean age showed a close resemblance, at 41.0 (*p* = 0.8), while 42% were females (*p* = 0.05). No further data for non-participating patients were available, but respondents’ baseline MCS scores were 28.0, compared with 30.0 among patients who completed only the baseline survey (*p* = 0.2).

## Discussion

The patients in the intervention group reported better satisfaction and mental health status at discharge than did those in the comparison group, albeit with small to moderate effect sizes.

Our finding of improved patient satisfaction corroborates the results of similar studies. In their longitudinal study of an IPE programme in a community mental health service, Carpenter et al. (2006) show similar results, albeit with small to medium effect sizes [21]. Moreover, our satisfaction score was similar to that of a cluster-randomized controlled trial (CSQ-8: 25.3) investigating the clinical effectiveness of collaborative care for depression in UK by Richards et al. [27]. Their controlled trial also found that patient satisfaction correlates positively with health status [27].

Although our study found improved patient satisfaction in the intervention group, the effect was smaller than expected, and its clinical significance is difficult to ascertain. However, a Cochrane review [34] by Papageorgiou et al. (2017) investigating interprofessional communication skills training for professionals working with severely ill mental patients found no difference between satisfaction scores for the intervention group and the comparison group (using CSQ-8).

We speculate that it is the increased focus on the patient-centred approach, implemented in the IPE unit that cause the improvement in mental health score, as active participation in own care is known to improve outcomes [35]. The usefulness of interprofessional collaboration in the field is well recognized, due to its capacity to provide and coordinate a variety of responses to patients’ complex healthcare needs [2].

Team-based care is a growing trend in mental healthcare delivery, where it is found to offer significant benefits for patients, ranging from more informed decision-making for complex conditions to improved access and reduced costs [2, 11]. However, its impact remains under-researched; Wen and Schulman’s (2014) systematic review thus found inconsistent results as to the effectiveness of team-based care and patient satisfaction [11]. Wen and Schulman point to the high or unclear risk of bias and the incomplete reporting of outcome data as examples of the poor trial quality evidenced in the studied literature [11].

In terms of psychological distress (K10), we identified no differences between the groups. This result corroborates the work of Carlier et al. (2014), who compared outcomes after an interdisciplinary re-employment programme among persons with mental health problems with standard care outcomes [36]. The pre- and post-K10 scores were slightly higher in our intervention group (31.5–29.4) than in Carlier’s intervention group (28.8–28.0), findings which we ascribe to the fact that theirs were primarily outpatients. Similar results were found in other studies assessing mixed groups of mental health inpatients and outpatients [29, 37].

The quality of evidence is critical to advancing our understanding of ways of improving interprofessional clinical training. However, studies undertaken in mental health contexts have so far produced limited results [2, 7]. Barnes et al. conducted a five-year evaluation of an IPE post-qualifying programme in mental health in the UK, measuring the outcomes of partnerships with patients. Whereas the patients improved in terms of social functioning and life satisfaction, their mental health status was unaffected [15]. Similarly, in their study of in-service IPE among community mental health teams, Reeves and Freeth [5] showed that while the educational input was well received, wider success was elusive, as already agreed plans for collaboration were not implemented. Moreover, no improvement in patient outcomes was reported.

Our study adds to the emerging international literature regarding interprofessional training and collaborative care. The documented improvements in self-reported mental health status and patient satisfaction corroborate previous findings [27, 38]. Further investigation, using e.g. RCT studies, are needed to study the effectiveness of IPE interventions.

### Limitations

The study design enabled analysis of changes in patient-reported outcomes for both the intervention group and the comparison group. However, some limitations should be acknowledged. As we did not observe the professionals or the students in interaction with patients, we are unable to conclude definitively on their actual behaviour in the interprofessional collaboration process. The analyses were not conducted as intention-to-treat with imputation of missing data. However, the two groups’ dropout rates were similar, and there is no indication of a dropout bias between the two wards. In order to fully explore the dynamics of an interprofessional approach in future research, conducting qualitative research study among already established interprofessional mental healthcare teams is likely to further elucidate the field. Owing to the nature of the study design, we were unable to randomize and blind patients to treatment groups. The referral procedure from the emergency ward to the two wards was not deemed to introduce a selection bias as it was determined by patients’ home address, and completely independent of the study hypothesis and standard for IPE studies of mental health patients [14, 15, 21]. Furthermore, the catchment areas of the two groups of patients were similar both in terms of socio-economic characteristics and living in a mixed urban and rural district.

### Implications for practice and future research

There is growing evidence in support of the need for and benefit of involving all healthcare professionals and patients in the planning, implementation, and evaluation of treatment [2, 14, 27]. The interprofessional training unit is a notable arena for the delivery of interprofessional care and education allowing students of the diverse fields of medicine, nursing, physiotherapy, and social care to collaborate on the delivery of care. IPE is already well established in other specialties and enjoys increasing global appeal [39, 40]. Our findings indicate that an IPE intervention may be beneficial for patient experience of the service. Although our study demonstrated positive effects, we cannot predict the long-term impact on patients. As the impact and sustainability of IPE initiatives in clinical settings are important to all parties involved [24], we believe that a longitudinal design is amenable to future exploration of such outcomes. We also recommend data collection strategies to provide insight into how IPE leads to change in healthcare processes and patient outcomes as research to date has not sufficiently addressed these critical issues.

## Conclusion

In summary, the patients in the intervention group scored higher on mental health status and satisfaction in comparison to the conventionally treated patients. No differences were found between the two groups in terms of psychological distress.

These positive findings add to the growing evidence in support of the claim that IPE training units are capable not only of creating unique and valuable environments for experiential IPE, but also of improving patient outcomes. However, as only a few IPE interventions have involved patients, and even fewer have taken place within the practice setting, further study of the processes as well as the long-term effects of IPE in mental healthcare is needed.

## Supplementary information

**Additional file 1.**

## Data Availability

All supporting data are included as additional files.

## Abbreviations

IPE: Interprofessional education
SF-36: Short Form Health Survey
K10: Kessler Psychological Distress Scale
CSQ-8: Client Satisfaction Questionnaire
